# The College of Nursing

**Published:** 1919-07-05

**Authors:** 


### THE COLLEGE OF NURSING

**(Limited by Guarantee).**

### The Manchester Conference.

### THE EDUCATIONAL VALUE OF LOCAL CENTRES.

Following on the paper read at the College Conference on June 19, in the Chemistry Theatre of the Manchester University, by Miss Brown, R.R.C. (Newcastle), on "Local Centres and How Their Usefulness may be Developed," reported in last week's issue, pp. 333-4, **Miss Milne** (Sister at the South Manchester Hospital, West Didsbury) spoke on the same subject. Many nurses, she said, asked what the College was doing. They had an idea that the College was something abstract, and the only thing they ever knew of its having done was the taking of a guinea which they did not want to part with —(Laughter). Local centres, too, they had heard of as bodies who asked for half-crowns. It sounded so simple. You paid a guinea to one place and half-a-crown to another, and then sat down and waited for the millennium—(Laughter). Automatically, shorter hours, more money, better training, State registration, and heaps of other nice things would come along. But don't let them be misled. Not one of these things would happen without a lot of work and trouble, and every individual nurse had as much right to do some of that work as her next-door neighbour —(Applause). The College was very much of a reality, and needed not only guineas but volunteers for all kinds of work. If they all did the work which was nearest and best suited to their individual capabilities they would, in this way, be hastening forward the millennium. How could the local centres give the best possible value for the half-crown subscription fee? First, by post-graduate lectures. There was always something to learn about drugs, medicine, and surgery, no matter how well-trained one might be. One's only means of keeping up-to-date was by hearing specialists lecture on their own pet theories.

A good course of lectures in the winter, with a few social meetings in the summer, would do very well for some people, but that was not all they wanted. They had heaps of other interests and hobbies which they must have time for if they were to retain any social gifts and keep their company of the kind which was sought after. The education of nurses had always proceeded along a certain groove, and had clung strenuously to those things which were supposed to be essentially nursing subjects, and there were such a number of those select subjects to cram into an all-too-short three years that the end of nursing training meant the end of an inclination to study. The result was a somewhat curious development. One side was highly trained and wonderfully technical, and the other was dull, hopeless apathy. The power to alter this state of things scarcely lay with the training-school, but it certainly lay with the local centres. One thing suggested itself very insistently to her—the need for a teacher of "how to study." Theoretical training would not present one-half of the difficulty if the best ways of absorbing knowledge could be made clear. She had seen nurses, many times, particularly just before an examination, painfully and unwillingly going into the study with a huge pile of books to spend a half-day in "swotting." In about half-an-hour, with very few exceptions, all the books were closed, and the nurse was far away in dreamland—(Laughter)—dreaming probably that she had passed with honours—(Laughter). But it was a very miserable nurse who came into the examination-room after such a preparation well provided with all the elements of failure. If some kind person could only have shown how odd ten minutes could be used for memorising during tram rides and tucking in beds, no half-days would be wasted, and that horrible sick feeling when the questions were first read over would be minimised, if not done away with entirely.

#### The Social Equipment of the Nurse.

Miss Milne proceeded to illustrate the poverty of a nurse's conversational powers, the gradual narrowing down of her interests, and the letting slip of early acquired social accomplishments. As a remedy she suggested that visits to the opera, to art galleries and libraries be arranged, and that later on an evening be given over at the centre to a paper written on a selected book, opera, or picture, when enthusiasts could air their own views. A library should form a large part of local centre property, and here, again, she would banish the technical in favour of the more interesting. If books were well selected they could be instructive in a subtle way. A nurse might be neither musical nor fond of reading, but she usually had some interest—photography, tennis, swimming, cycling, walking, dancing, gymnastics, and a hundred and one things that could be made the subject first of a happy meeting, and later of a paper or debate. An inter-hospital debate would prove interesting, instructive, and highly amusing.

A club could be run on the most approved lines, offering a welcome to all kinds and conditions of nurses. Here the library could be housed, together with the pictures and hangings selected by the artistic members. There could be rest-rooms, reading-rooms, refreshment-rooms, and, afterwards, bedrooms where nurses travelling would be sure of accommodation. Local centre meetings were nearly always for members only. Miss Milne pleaded for the probationer nurse, who was, after all, the nurse of the future, and with her lay the traditions of the profession. Opponents of the College said that many nurses had been coerced into joining the College—(Hear, hear)—that they did not understand anything about it, and that, through fear of their matrons, they must be members. At present this could be very true, and it was one of the biggest propositions for the local centres to tackle.

#### A Surfeit of Ideas.

The discussion was opened by Miss E. M. Musson, matron of Birmingham General Hospital. She thought if they were to do all the things proposed by the lecturer they would have to get busy. (Laughter.) She was suffering from a surfeit of ideas, and so found it difficult to criticise. They all felt that some combination of training-schools was necessary if they were to be run satisfactorily. Whether it would be the duty of the local centres to launch such a big thing as that she could not say. It would seem to rest with hospitals to set up and run schools for probationers. The giving of lectures was a piece of work that might well be done by the centres. Exchanges of probationers between hospitals would be simpler when a uniform curriculum was introduced, and when they had sister-tutors. Under those

**The College of Nursing**—(*continued*).

conditions she saw no insuperable barrier in the way of exchanges between hospitals. It should not be their aim to turn out nurses practised in theory who were not practical nurses. (Applause.) There was one point in Miss Milne's interesting and amusing address from which she differed very strongly. Miss Milne said a probationer might be taught to memorise part of her lessons while tucking up a bed. She (Miss Musson) always tried to impress upon her probationers, however busy they might be, to give their whole attention—their thoughts as well as their hands—to the patients whose beds they were making. (Applause.) Local centre magazines was a very ambitious thought of Miss Brown. Nothing was more difficult than to run a small magazine, but she hoped the College would have a magazine of its own, and that members would support it not only by subscribing to it, but by sending in papers. (Hear, hear.) The centres were going to be useful, as Miss Milne had said, from the educational and the social points of view, and they had already done a great deal. Through their medium nurses of all branches had been brought together as they had never been brought together before, so that they were brought to a realisation and an appreciation of each other's difficulties. These difficulties could be brought up at the centres, and after being thoroughly threshed out could be brought before the Council of the College, and something might there be done to alleviate hardships and remove difficulties. She hoped the local centres would not devote too much time to local detail. They should remember that local centres were part of the College of Nursing. As it was, there was too much tendency to say, "Do you belong to the Manchester Society?" or "Do you belong to the Birmingham Society?" They were all parts of the one great whole. (Applause.) They had also to remember that they had to work not only for the good of the profession, but for the good of those for whom they existed.

Main Objectives of Local Centres.

**Miss R. Cox-Davies,** Matron of the Royal Free Hospital, London, wished to emphasise very strongly indeed the reference by Miss Musson to the necessity for whole-hearted unity between the local centres and the College of Nursing. The one object of the local centres until the College was established ought to be the building up of the College, getting it into a good healthy condition, and supporting it in every possible way, even at the risk of the centres not developing quite so grandly, usefully, or quickly for the nurses of a particular area as had been hoped. It was only by building up the College as the great headquarters that they would be able to do all they wanted to do for the profession. (Applause.) A great deal was being done in studentships and courses, and she hoped that this idea would develop to such an extent that it would be possible before long, instead of having the courses arranged for them by non-professional people, to have them arranged by one of their own profession holding a lectureship at one of the Universities. Let them see that they get the right women in posts for the development of the profession, and let them get the right things taught to the probationers in the schools, and not cram their heads with mere theory. Let them, in fact, get the right women with the right hands and right hearts. (Applause.)

**Miss A. Hughes,** late General Superintendent Q.V.T., thought the effect of the new spirit as visualised in the local centres and the College would be to raise the standard of the profession, and to let the public see what nurses really were.

**Miss Seymour Yapp,** Matron of Ashton-under-Lyne Poor-Law Infirmary, made a spirited and frequently applauded contribution to the discussion, but, unfortunately, her position near the rear of the lecture-room made her remarks difficult to hear in the vicinity of the platform. She advocated a broader outlook for nurses, and a keener cultivation of wider interests, and the social instinct. She knew how difficult it was to entertain nurses if there was no dancing or no whist. (Laughter.)

**Sir Cooper Perry,** who had now taken the chair (Sir Henry Miers having been called away to other duties), said if by any scheme of affiliation the lot of the smaller hospitals could be rendered easier and the profession improved he would welcome it heartily, but he thought the practical difficulties of affiliation were very great. Should the Local Centres succeed in solving those difficulties they would do a great service to nursing.

### THE IDEALS AND ETHICS OF NURSING.

The meeting then proceeded to the second session of the Conference, and **Miss A. W. Gill,** Lady Superintendent of Edmonton Royal Infirmary, read a paper on "Nursing Ethics." They were, she said, so much occupied to-day with questions of professional status, higher salaries, and less work, that there was a fear that the ethical side of nursing might be forgotten. After all, wages, and the questions relating thereto, did not form the main-spring of their life and work. Character was of the first importance in a nurse, and she had always held that a nurse must be a good woman. (Applause.) Conduct and habits counted for so much in dealing with the sick that it was necessary to have definite instruction given on those subjects during training. Teaching should be by precept and example from the commencement of the training period. Many probationers, when first they entered the hospitals, were ignorant and impressionable, and had not had time to think out the problems of life for themselves. Those four years of the probationary period, therefore, had great influence in moulding the character of the nurse.

A Profession: Not a Trade.

If they wanted nursing to be a profession and not a trade they must have ethical standards. (Loud applause.) As a class they must be known by these standards: it was no use having standards if they were not known by them. If they wanted nursing to be an art—and it was a fine art—(Hear, hear)—they must find their chief pleasure and interest in their work. (Applause.) It was an unfortunate modern tendency in all work for everyone to find pleasure outside his or her work. The work ceased to be a pleasure and became a necessary evil for the purpose of providing a livelihood. This might do with a trade, but it would not do with an art. There was certainly a tendency to fall away from standards, and much criticism had been levelled at nurses.

They would notice in novels in which nurses appeared that it was seldom the technical side of the nurses which was attacked, but it was the personal side—their conduct or habits, something which could have been cured in their training. They erred, apparently, on the ethical, and not on the technical, side of their work. She made

**The College of Nursing**—(*continued*).

a plea for a more systematic teaching of ethics for nurses, and a little more outspokenness on the part of their seniors. Do not, concluded Miss Gill, preach at them. Anything in the nature of a sermon passes on their heads and leaves them cold. With nurses, as with other human beings, the best will always make an appeal. (Applause.)

A Little Understood Profession.

**Miss A. Lloyd Still,** C.B.E. (Matron of St. Thomas's Hospital, London), supplemented Miss Gill's address in a paper upon the same subject. She began by saying that surely no profession as an organised body was so incomprehensible to the outside world, and its workings so little understood, even to one's own immediate circle, as nursing. The well-meaning but ignorant public had taken up the nurses' cause, voicing their so-called "grievances," stimulated by the discontent in their ranks. There was great danger that the sense of service would be sacrificed in the precipitate and drastic readjustments demanded by the ill-informed public. There was much need for reform; but it had been going on steadily for some years, and the fact that grievances were being voiced so loudly by popular clamour was evidence enough that they were well on the way to redress. The sense of service was already weakened.

The public mind would rank nurses with the mechanical labourer who spends his life at some soulless task of repetition, not with one who, for the sake of a profession that calls for head, hand, and heart, will give his utmost and his best, deeming his richest reward the soul satisfaction that comes in exact proportion to the spirit and effort expended. The trade-union spirit was death to the nurse's calling. It was impossible that nurses worthy of the name could leave the sick and suffering uncared for while they settled some grievance of their own, real or fancied, by t e trade-union method of a strike. Yet the public in their ignorance would bring this about if they had the handling of nursing affairs. One much-lauded remedy was *living out*. A bachelor life might have attractions for some if their salary could be supplemented from home; but otherwise it had many hardships, as the trained nurse knew who set up for herself. The training nurse would find it difficult to make ends meet. She was out to make herself proficient and to be able to command adequate remuneration in the future for her trained skill; therefore, she must keep herself in sound health, and in a large institution this was made possible without any *conscious* effort on her part, and she was provided with many comforts unobtainable in "diggings."

Selfless Enthusiasm.

The public demanded short hours for nurses, such as were demanded, and rightly, by the mechanical trades, where there was no scope for living interest, for initiative, but only the dead level of a soul-killing occupation. But nurse's work was of varied and absorbing interest of the most satisfying kind, with the constant refreshment of effort well spent. Did any service with aims like theirs measure its labour by time? They all knew that the hours were too long; but they were speedily being revised, and were shorter now than five years ago even. It was well to remember they resulted from the enthusiasm of their predecessors and their selfless devotion, besides the fact that the public have not made it possible, by adequate financial assistance, to increase the nursing staff of their voluntary and parochial institutions.

Disadvantages of the Eight-hour Day.

The eight-hour shift would be greatly to the patient's disadvantage. It would drive out of their midst the ideal ward sister—the pride of British hospitals—for no woman who loved her patients and her work would consent to the division of her service of love. The British nurse had always said: "Patients first." Was the nurse of the future to take a lower standard and look on the patient as merely her training material?

It was the untiring self-sacrifice so willingly given that was the true healing art. Is the nurse in training to be debarred from gaining full experience of the wards and from deriving full benefit from the educational course in the three short years allotted to her because she is bound to eight hours and no more? Would nurses see a longer period of training in force to make good the curtailed day and its loss of experience?

When it came to more pay, then the nursing profession was at one with the popular voice, and hoped the cry was an earnest of substantial increase in the generosity of the public, for only by that means could adequate remuneration be supplied. Long had matrons striven to improve the conditions of the trained staff; but the hospital funds had other claims; the means were not forthcoming for those who served the sick public, either in voluntary hospitals or in the Poor-Law institutions—rather less so in the latter, where the State was the benefactor. It was not to be expected that the probationer nurse should be given a salary irrespective of the value of her training, which was in many cases costly, and tended to increase. Till after her third year she was hardly a valuable hospital asset. Surely the exploiters of nurses were those who obtained their *trained* services for a minimum wage. The public did not seem to be able to distinguish the difference between the training and the trained nurse. What private nurse, if trained under these conditions, would consent to give more than a third of her day to her patient, and in the same measure her interest?

The Absence of a Sense of Responsibility.

If nurses did but preserve the sense of vocation—the spirit of service—they need fear little harm from changes forced from outside; adjustment would come in time. But there was a disquieting lack of the sense of personal responsibility, a growth of individualism and self-seeking in their midst, that boded ill for future development. Lack of discipline was a feature of the present day, especially noticeable now, when all nurses held stable was in the melting-pot. Less disciplined in home life than formerly, the probationer found restrictions more irksome. The ideal was to have no rules; but only self-discipline would make this possible, and yet secure at the same time the common good of the community. Every thoughtless act or deed of selfish gratification on the part of a nurse reacted to the discomfort or deprivation of her colleagues. To the disciplined woman there were no real hardships in hospital life, and as her experience grew she realised the inestimable value of an ordered life and the saving of energy, with increase of power, resulting from the routine habits inculcated in her training days. The speaker here made an eloquent protest against the mistaken sympathy excited by nurses' "menial work." The glory and dignity of their calling suffered outrage by such a term. Observation, thoroughness of detail, neatness, and finish must be practised and perfected with ordered, quiet swiftness on inanimate material before the 'prentice hand and mind were allowed the sacred task of handling the sick; and to call any work menial in such a relation was sacrilege.

**The College of Nursing**—(*continued*).

Training the Intelligence.

A nurse should not be overburdened with mechanical work, essential as it was in the formation of good habits, and those who were responsible for the training of probationers should ever bear in mind they were there to be trained in intelligent observation and in the skilled care of the patient, and were not to be regarded only as a pair of hands.

Difficult days were ahead in the training of war probationers. The freshness was gone, the humility was often lacking, the outlook was critical—material not easily moulded to traditional standard. But where these half-trained will at the outset deliberately cast to the back of their minds for the time being all their previous experience, and bring to their systematic training a blank sheet, success should be more speedily achieved, as in their second and third years they fit into place former knowledge now duly proportioned to the whole.

If to some her words might seem too stern, her foreboding too serious, it was love that had made her take that tone—love of the true British nurse, love of a great heritage, love of their great mother-foundress, Florence Nightingale, who gave them this heritage. . . .

The Nurse's Halo.

**The Dean of Manchester** (the Very Rev. Dr. Swayne), speaking as a member of the public, said they were apt to surround the nurse with a halo, and to demand from her almost superhuman powers, and he had no doubt that many of them, if they were conscious of that attitude, were rather apt to resent it. He would say to them in all seriousness not to resent altogether this attitude of the public, because their profession was a very noble one, and it was only noble women who could properly officer this great and noble profession. He was going to say something which had been said to him by nurses, and which did not arise out of his own observation at all. Nurses had told him they had frequently to be on their guard against the tendency to get "hard." If such was the case it had not been revealed to him, and one of the happiest things in the hospital he visited in London in the capacity of honorary chaplain was the story told by patients of extraordinary kindness done by everybody, specially by the nurses. But if the nurses said there was a danger of growing "hard," there must be something in it. Frequent contact with human suffering made one get used to it. Doctors, clergy, and nurses could not possibly do their day's work if they continued as sensitive to human suffering as they were when first they took up their duties. It did not affect them in the same way, but they could be more efficient. They must watch, however, that the will to serve did not weaken. If they entered into their work with the high ideal of the will to serve, though they did not feel the early sensitiveness in the presence of suffering, they would never get "hard." Nurses must always be something of psychologists, and they must realise the great part played by personality in their work.

On the call of Miss Lloyd Still the cordial thanks of the Conference were extended to Miss Sparshott and the staff of the Manchester Royal Infirmary for their hospitality. Miss Musson supported the vote of thanks.

Earlier in the day visits were paid to the Royal Infirmary, a cotton mill, Chetham College, and the aerodrome at Alexandra Park.

### THE COLLEGE OF NURSING (Limited by Guarantee).

### LONDON CENTRE.

We are requested to announce that any communications regarding the following fixtures should be addressed to the Hon. Secretary, the London Centre's Club, 7 Henrietta Street, W. 1:

Tuesday, July 15, 4—7.—Miss Brown, "At Home," King's College Hospital.

Thursday, July 24, 11 a.m.—Visit to Messrs. Cook & Sons, soap specialists, Bow.

### LIVERPOOL CENTRE.

Friday, July 4, at 7 p.m.—A members' meeting will be held at the Royal Infirmary to discuss the establishment of a Chair of Nursing at King's College for Women or some other university, to be supported by the College of Nursing.

### BRIGHTON AND HOVE CENTRE.

A Residential Club.

It will be good news to all the members of this Centre to know that a Club has been opened, as a College of Nursing Club, at No. 2 Old Steine. In addition, this Centre is to have its Residential Club. This has been made possible through the kindness of one of the members of the Finance Committee, who has kindly placed at the Centre's disposal for this purpose from October 1 next a house, No. 45 Marine Parade, Brighton. Situated a little to the east of the Palace Pier, on the sea front, and with an extensive and uninterrupted view over the sea, it is within a few minutes' walk of the theatre, Hippodrome, etc., and a very short distance from the shopping centre. The house will accommodate about fifteen residents in addition to the domestic staff, has a beautiful and spacious drawing-room, a large room in front, which will probably be used as a lounge and smoking-room, and an ideal room, also in front, which will be used as a refreshment-room. The bedrooms chiefly take the form of cubicles, but they are all separated by a high wooden partition, and each has a door provided with a lock and key. Electric light is fitted throughout, there is a plentiful supply of hot water and bathrooms, and contains an excellent kitchen and cooking arrangements. The scale of charges has not yet been fixed, but it will be made as reasonable as possible.

Until this Club is opened College nurses in Brighton will be welcomed at the Club in 2 Old Steine, which, however, is not residential.

### SISTER-TUTOR STUDENTSHIPS.

The examinations for the College of Nursing Sister-Tutor Studentships were held on Saturday morning and afternoon, June 28. There were thirty-seven examinees, of whom thirty-two sat in London, three in Edinburgh, two in Dublin, and one in Belfast

### MEETING AT NORWICH: NEW CENTRE TO BE FORMED.

About 200 nurses were present at a meeting at the Norfolk and Norwich Hospital on Saturday, June 28, when Miss Cowlin, the Organising Secretary of the College of Nursing, spoke on the work of the College and the value of Local Centres. The chairman of the hospital, Sir Eustace Gurney, presided, and he was supported by Mr. Horsfall, a former chairman. Miss Cowlin emphasised the value of the Centres in carrying out the policy of the College and the importance of their co-operation with headquarters. This was necessary if the individual nurse is to assist in the management of her own professional affairs. The watchword of the College should be efficiency, and it was in the hands of the College members to show the public the difference between a College nursing sister and the semi-trained and unskilled worker. A resolution proposing that a Centre of the College for Norfolk and Norwich be established in Norwich was carried unanimously, and an Executive Committee was appointed to meet on Saturday, July 12, at the Norfolk and Norwich Hospital.

